# Expression and Change of miRs 145, 221 and 222 in Hypertensive Subjects Treated with Enalapril, Losartan or Olmesartan

**DOI:** 10.3390/biomedicines9080860

**Published:** 2021-07-22

**Authors:** Giuseppe Mandraffino, Alberto Lo Gullo, Maria Cinquegrani, Angela D’Ascola, Davide Sinicropi, Egidio Imbalzano, Giuseppe Blando, Giuseppe Maurizio Campo, Carmela Morace, Clemente Giuffrida, Salvatore Campo, Giovanni Squadrito, Michele Scuruchi

**Affiliations:** 1 Internal Medicine Unit, Department of Clinical and Experimental Medicine, University of Messina, 98122 Messina, Italy; mariacinquegrani@gmail.com (M.C.); sinicropidavide@gmail.com (D.S.); eimbalzano@unime.it (E.I.); giuseppe.blando91@gmail.com (G.B.); cmorace@unime.it (C.M.); gsquadrito@unime.it (G.S.); 2 Lipid Center, Internal Medicine Unit, University of Messina, 98122 Messina, Italy; mscuruchi@unime.it; 3 Laboratory of Clinical Biochemistry, Department of Clinical and Experimental Medicine, University of Messina, 98122 Messina, Italy; angela.dascola@unime.it (A.D.); gcampo@unime.it (G.M.C.); 4 IRCCS Neurolesi Bonino Pulejo, 98123 Messina, Italy; clemente.giuffrida@irccsme.it; 5 Laboratory of Molecular Biology, Department of Biomedical and Dental Sciences and Morphofunctional Images, University of Messina, 98122 Messina, Italy; scampo@unime.it

**Keywords:** microRNA, atherosclerosis, arterial hypertension, arterial stiffness, cell programming

## Abstract

miR profile could be associated to CV risk, and also to prognosis/outcome in response to therapeutic approach. We aimed to evaluate if anti-hypertensive drugs enalapril, losartan or olmesartan have effects on monocyte miR profile in essential hypertensives without target organ involvement. For this purpose, 82 hypertensives and 49 controls were included; we evaluated SBP/DBP, lipid profile, glucose, CRP, fibrinogen, arterial stiffness indices (PWV; AIx), and cIMT at baseline (T0) and after 24 weeks of treatment (T1). Subjects with LDL-C ≥ 160 mg/dL, TG ≥ 200 mg/dL, BMI ≥ 30, and other additional CV risk factors were excluded. Patients who were prescribed to receive once-a-day enalapril 20 mg, losartan 100 mg or olmesartan 20 mg were eligible for the study. At T1, we found a significant improvement of SBP (−18.5%), DBP (−18%), HDL-C and LDL-C (+3% and −5.42%), glucose (−2.15%), BMI (−3.23%), fibrinogen (−11%), CRP (−17.5%,), AIx (−49.1%) PWV (−32.2%), and monocyte miR expression (miR-221: −28.4%; miR-222: −36%; miR-145: +41.7%) with respect to baseline. miR profile was compared to control subjects at baseline and at T1. We found some little difference in the behaviour of the three treatments on some variables: olmesartan was the most effective in reducing fibrinogen, DBP, CRP, and AIx (−13.1%, −19.3%, −21.4%, and −56.8%, respectively). Enalapril was the drug more significantly increasing the expression of miR-145. In conclusion, enalapril, losartan and olmesartan are effective in improving mechanical and humoral factors associated to AS and atherogenesis. These drugs appear to be able to modify miRs 221/222 and miR-145 expression in drug-naïve hypertensives, making it closer to that of control subjects; additionally, this provides a good blood pressure compensation, contributing to slow the progression of vascular damage.

## 1. Introduction

A growing number of studies suggest the importance of non-coding genome in the modulation of a number of biological processes, including regulation of gene expression and epigenetic modification, and in the development of different diseases. Non-coding genome accounts for about 98.5% of the entire gene pool; however, it is widely transcribed in regulator non-coding RNA (ncRNA) [1,2]. A subset of ncRNA is represented by small RNAs with less than 30 nucleotides. The widely studied microRNAs (miRs) act as post-transcriptional regulators of gene expression by degrading mRNA or inhibiting messenger RNA translation. Due to their function, miRs are implicated in a plethora of biological processes including cell development and proliferation, lipid metabolism, angiogenesis, and vascular homeostasis [3,4]. Thus, dysregulated expression of miRs has been seen to associate with pathological conditions, such as tumorigenesis and cardiovascular disease (CVD) [5,6,7,8,9]. Recently, Jayaseelan suggested a list of miRs potentially implicated in arterial hypertension [10], and Tan et al. collected the most recent evidence involving miRs in the development of essential hypertension and its potential as biomarkers [11]. Moreover, the latest news has been provided in very high-risk diabetic people, involving miR-33 in the pro-inflammatory and pro-coagulable state of coronary thrombi in hyperglycemic STEMI patients [12], and suggesting miR-24 expression to be associated to MACE during a 2-year follow-up in prediabetic patients with asymptomatic carotid artery stenosis [13].

Human miR-221 and its paralogue miR-222 are important players in the vascular environment, as they influence angiogenic properties of endothelial cells (ECs) and phenotypic changes in vascular smooth cells (VSMCs) [14,15]. Essential physiological vascular processes, such as angiogenesis [16], neointimal hyperplasia and vessel wound healing [17], and also vascular aging [18], seem to be regulated by miR-221/miR-222. Furthermore, these miRs are implicated in a variety of vascular-related pathological mechanisms including tumour angiogenesis [19], atherosclerotic inflammation and vascular remodelling [20], fibrosis [21], vascular calcification [22], cardiac hypertrophy [23], angiotensinII-dependent hypertension [24], and diabetic hyperglycaemia-induced endothelial dysfunction [25].

miR-145, one of the most expressed miRs in VSMCs [26,27], has been involved in the regulation of calcium signalling [28], contractile differentiation [29], cell proliferation and migration [30]. Furthermore, miR-145 expressed from VSMCs has been shown to modulate the angiogenic and vessel stabilization properties of endothelial cells (ECs) in a paracrine and endocrine manner [31].

In recent studies, we found changes in miR-221/222 expression in CD34+ circulating progenitor cells (CPCs) from hypertensive patients with isolated arterial stiffening (AS) or with both carotid intima-media thickening and left ventricular hypertrophy (LVH), and also an association of miR-221/222 with CD34+ cell number and ROS levels [8]. We also observed that a 6-month treatment with olmesartan medoxomil, an angiotensinII-type1 receptor (ATR1) blocker (ARB), is effective in reducing miR-221/222 expression and ROS levels in CD34+ CPCs from hypertensive patients presenting with left ventricular hypertrophy, likely by modulating ATR1 activity [9].

Since miR-145 is highly expressed in VSMCs, but almost undetectable in ECs [32], we chose to study circulating CD14+ monocyte cells in order to evaluate the behavior of this miR in arterial hypertension at baseline and after an anti-hypertensive treatment was started.

Briefly, in the present study we evaluate: (a) miR profile in subject and controls; (b) potential effects of the anti-hypertensive drugs enalapril, losartan and olmesartan on CD14+ monocyte miR profile in hypertensives without hypertension-mediated organ disease; (c) potential associations between clinical parameters and miR profile.

## 2. Materials and Methods

### 2.1. Subjects

**Figure 1:**
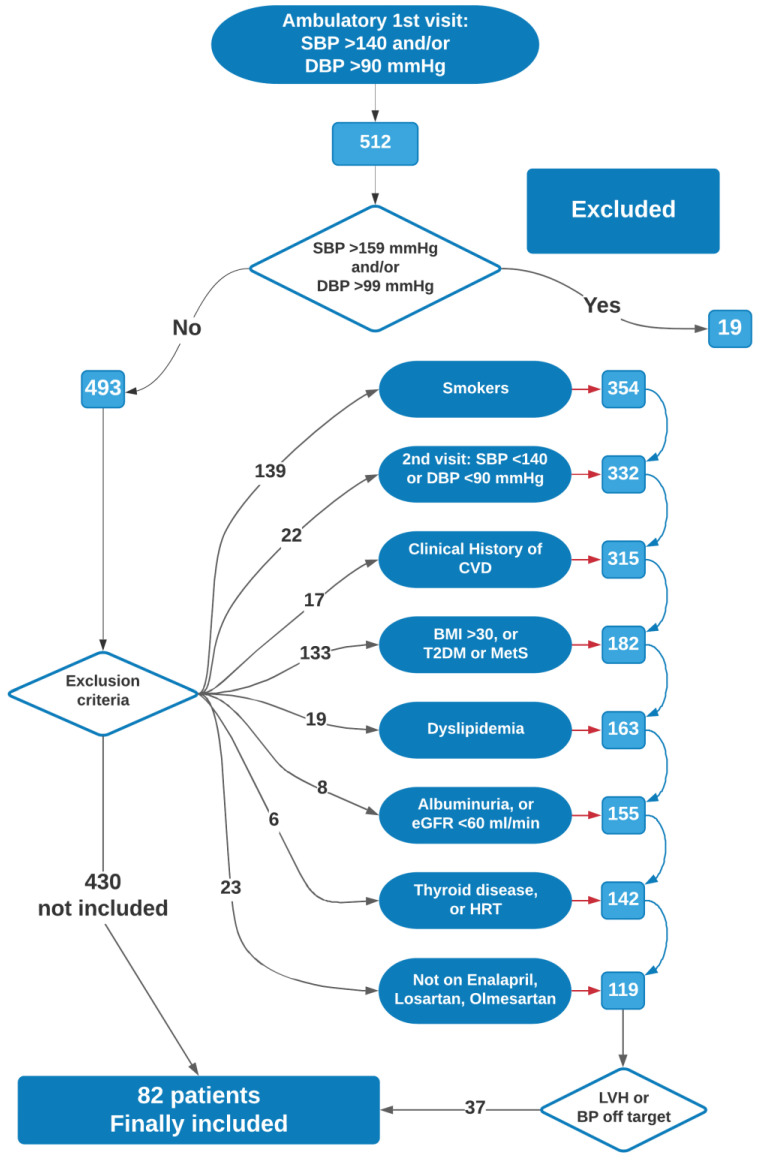
Selection flow of the final study population. Figure 1 represents the flow diagram for patient inclusion/exclusion path. (flow diagram was drawn by Lucidchart©, Lucid Software Inc., 2021; www.lucidchart.com; access date: 31 May 2021).

The data used for this study were obtained from the medical records filed at the Hypertension Clinic of our Department; according to the aim of the study, we selected only non-smoker hypertensive patients, with stage 1 hypertension and no ultrasonography signs of LVH, prescribed to start a monotherapy with enalapril 20 mg, or losartan 100 mg, or olmesartan 20 mg. Figure 1 shows the selection flow of the final study population. The selection started from 512 (M/F = 272/240) consecutive outpatients referred for the first time to our clinic between November 2014 and April 2015 (newly diagnosed hypertensive outpatients) with at least two aliquots of stored frozen serum. Essential hypertension was diagnosed as systolic blood pressure (SBP) ≥ 140 mmHg and/or diastolic blood pressure (DBP) ≥ 90 mmHg, in repeated home measurements, further confirmed by office measurement. Smokers were immediately excluded. Patients with office SBP ≥ 180 mmHg and/or DBP ≥ 110 mmHg, or with SBP < 140 mmHg, were also excluded. Patients with a clinical history of CVD or alcohol consumption, with body mass index (BMI) ≥ 30, diabetes mellitus, low-density lipoprotein cholesterol levels (LDL-C) ≥ 160 mg/dL, triglyceride levels (TG) ≥ 200 mg/dL, urinary albumin excretion ≥ 30 mg/24 h, or with thyroid, kidney or liver diseases were sequentially excluded from the analysis. Women taking hormone-based therapy (HRT) were also excluded from the study. In accordance with our current clinical practice, secondary hypertension was systematically excluded, and complete clinical and laboratory examinations were performed and integrated with carotid echo-Doppler scan implemented by AS evaluation and with echocardiography study; LVH was excluded by transthoracic echocardiography; secondary hypertension was excluded, as were comorbidities, co-treatments, and target organ damage. Clinical and instrumental examinations were completed within two weeks from first visit. Behavioural norms (caloric and salt intake restriction, weight loss, attitude to aerobic physical activity) were prescribed for at least six weeks. Blood samples were collected at time of diagnosis; no patients or controls were taking medications. BP was measured using a validated digital oscillometric device, Omron 705IT (HEM–759–E) (Omron Corporation-Healthcare, Kyoto, Japan). Three measurements performed with intervals of at least 2 min were then averaged.

All analyses were performed on a venous blood sample taken at the medical centre. Total cholesterol (TC), TG, high-density lipoprotein cholesterol (HDL-C), glucose and fibrinogen were measured by routine methods. Low-density lipoprotein cholesterol (LDL-C) was calculated using the Friedewald formula. High-sensitivity C-reactive protein (HsCRP) was determined using an immunoturbidimetric latex assay kit. A blood sample was also obtained to evaluate miR expression (qPCR).

Once laboratory screening was completed, after six weeks of non-pharmacological management patients having SBP ≥ 140 mmHg and/or DBP ≥ 90 mmHg started drug therapy; patients who had received enalapril 20 mg or losartan 100 mg or olmesartan 20 mg as antihypertensive monotherapy were then considered for the study; from clinical records, we analysed data covering the 24 weeks following the prescription (with office re-evaluation every 3–4 weeks). Patients were recommended to maintain behavioural norms also after they started drug therapy; weight was recorded and BMI calculated at each visit.

Briefly, we finally identified 82 patients (M/F = 47/35) who had completed the needed observation period (24 weeks) with no need of therapy modifications and who underwent the requested clinical/instrumental examination to be considered for the statistical analyses.

Forty-nine healthy subjects (M/F = 28/21) were also enrolled as control subjects from hospital personnel. All the hospital staff went through a detailed medical history, blood pressure measurement and thorough check of blood tests and, therefore, were identified as subjects in full health (health surveillance program).

### 2.2. Measurement of cIMT, Arterial Stiffness and LV Parameters

Carotid ultrasonography evaluation and AS assessment have already been described [8]. Briefly, semi-automated cIMT evaluation was performed using Aloka ProSound ALPHA10 with a 7–15 MHz linear array transducer; following ESC/ESH guidelines, we considered a mean cIMT ≥ 0.9 mm or plaque as carotid wall thickening. Augmentation index (AIx) and pulse wave velocity (PWV) were measured automatically by “eTRACKING” software as AS indices. According to the method chosen to assess AS indices, we did not use pre-fixed cut-offs to classify normal or abnormal PWV and AIx; since these indices are continuous variables, we considered PWV and AIx values as compared to the normotensive control mean. LV examination was performed following American Society of Echocardiography recommendations, using a Vivid-7 ultrasound system (GE Medical Systems, Horten, Norway) equipped with a cardiac M4S transducer. LV mass was determined with the area-length method, and the LV mass index (LVMI) was calculated as LV mass/body surface area (BSA) (g/m^2^) ratio. LVH was diagnosed as a LV mass index (LVMI) ≥ 102 g/m^2^ in men and ≥ 81 g/m^2^ in women [33].

### 2.3. CD14+ Cell Identification and Separation, miR Expression

miR-221 (NR_029635.1), miR-222 (NR_029636.1) and miR-145 (NR_029686.1) expression were evaluated in CD14+ peripheral blood mononuclear cells (PBMC), isolated from heparinized venous blood of population study (82 people) and controls (49), as already described [34]. All samples were processed immediately after collection. PBMCs were separated from other blood components by density gradient centrifugation using Lympholyte separation medium, and CD14+ PBMC were enriched by using the MiniMACS system according to the manufacturers’ instructions (Cedarlane, Burlington, ON, Canada; Miltenyi Biotec Inc., San Diego, CA, USA). Finally, the cell enrichment was validated by flow cytometry, confirming that at least 90% of separated cells were CD14+. CD14+ cell enriched samples were frozen immediately after processing and stored at −80 °C. After patient selection, samples were processed as follows (<180 days from blood drawn): for miRs’ expression study, total RNA was extracted from lymphomonocyte pellet using the Total Purification Plus Kit (Norgen Biotek Corporation, Thorold, ON, Canada), which is designed for DNA-free pre-miRNA recovery, according to manufacturer’s instructions. Total RNA was quantified at 260 nm using a spectrophotometer (BioMate3, Thermo Electron Corporation, Bedford, OH, USA); its purity was evaluated by the ratio of readings at 260/280 nm ≥ 1.8. cDNA was obtained from 1µg of total RNA, using the All-in-One miRNA First-strand cDNA synthesis kit (GeneCopoeia Inc., Rockville, MD, USA), which couples both poly (A) polymerase and reverse transcriptase in a buffer that allows the maximal performance of both enzymes. In such a reaction, the poly (A) polymerase adds poly (A) tails to mature miRNAs to generate poly (A) miRNAs. In the same reaction, the retro-transcriptase (m-MLV RTase, 8 U/μL), using a unique 2.4 μM oligo (dT) adaptor primer, reverse transcribes the poly (A) miRNAs into the corresponding poly (dT) cDNAs. The reactions were performed with the adding of 1 U/μL of RNAase inhibitor in a final volume of 25 µL, according to the manufacturer’s protocol, as follows: 65 °C for 10 min; cooling on ice for 1 min, 37 °C for 60 min; 85 °C for 5 min. The miRs’ expression profile, in both normal and diseased subjects, was independently measured by qPCR using a 7500 Real Time PCR System (Applied Biosystems, Foster City, CA, USA). After the cycling process, a melting curve analysis was performed to exclude unspecific PCR products.

The reactions were executed in triplicate using the All-in-one qPCR mix, that provides a SYBR Green based real-time PCR (GeneCopoeia Inc., Rockville, MD, USA), 50 ng of DNA template, 150 nM ROX Reference Dye and a couple of PCR primers (forward: 0.2 µM miRNA-gene specific primer; reverse: 0.2 µM Universal primer, that anneals the adaptor sequence at the 5′ end of the cDNAs) which had been pre-validated by GeneCopoeia Inc. Two different small nucleolar RNAs (SNU6 and SNU4) were used as internal reference genes. The reaction protocol was set as follows: 95 °C for 10 min; 95 °C for 15 s; 60 °C for 1 min. Steps 2 and 3 were repeated 40 times and the fluorescence acquisition was set at step 3. All Ct values reported as ≥37 or as N/A (not detected) were considered as not detectable, to avoid misleading results A dissociation step was added at the end of all reactions to assess the specificity of each result.

The normalized average values of miRs in samples from all control subjects were considered as the calibrator (1 × sample) and results were expressed according to the 2^−ΔΔCt^ calculation, as n-fold differences relative to calibrator (relative expression levels).

### 2.4. Ethics Statement

Written informed consent for the use of clinical data was obtained from all subjects in accordance with the Helsinki declaration; this observational study has been approved by the Ethics Committee of the University of Messina (prot. Number 07/15).

### 2.5. Statistical Methods

The Kolmogorov–Smirnov test was used to verify the distribution of the study variables. Since some variables had a non-normal distribution, and also given the relatively small size of our sample, we chose a non-parametric statistical approach. Baseline characteristics of hypertensive subjects were thus compared with control subjects by Mann–Whitney test, as were T1 values vs. controls; also, T1 vs. T0 changes in hypertensive cases were compared by Wilcoxon test. The mean difference of each variable at the two time-points was evaluated by the mean of the change of each patient, as mean relative Δ%, calculated as follows: (T1-T0)/T0 × 100. The mean change % over time was evaluated among the three groups (by Kruskal–Wallis test). Any difference in change % between T0 and T1 among the three treatment groups (according to the drug prescribed) was noted, and verified by pairwise two-by-two comparisons with Bonferroni correction. A two-tailed alpha of 0.05 was used to denote statistical significance. To perform statistical analyses, we used the SPSS statistical package, version 26.0 (Chicago, IL, USA).

## 3. Results

The study population consisted of 82 hypertensive subjects, selected as shown in Figure 1; Table 1 summarizes the baseline characteristics of the study population. There were no differences as regards age, BMI, lipids and glucose between hypertensive subjects and controls. SBP and DBP values were higher in hypertensive subjects, as were also fibrinogen and HsCRP (both *p* < 0.001). Although LVMI values were comparable between hypertensive and control subjects, AS indices and also cIMT were higher in hypertensive cases (all *p* < 0.001). miR-221 (1.9 vs. 0.97, *p* < 0.001) and miR-222 (1.89 vs. 0.93, *p* < 0.001) were significantly increased in hypertensive subjects, while miR-145 was reduced (0.60 vs. 1.04, *p* < 0.001).

After 24 weeks (T1) of treatment (enalapril or losartan or olmesartan), SBP and DBP values were significantly lowered (SBP: ∆ = −18.5% vs. baseline, *p* < 0.001; DBP: ∆ = −18 vs. baseline, *p* < 0.001) as were improved lipid profile (HDL-C: ∆ = +3% vs. baseline, LDL-C ∆ = −5.4% vs. baseline, both *p* < 0.001), glucose (∆ = −2.15% vs. baseline, *p* < 0.001), BMI (∆ = −3.23% vs. baseline, *p* < 0.001), fibrinogen (∆ = −11% vs. baseline, *p* < 0.001), HsCRP (∆ = −17.5% vs. baseline, *p* < 0.001), AS indices (AIx: ∆ = −49.1% vs. baseline, *p* < 0.001, PWV: ∆ = −32.2% vs. baseline, *p* < 0.001) and also miR expression (miRs–221: ∆ = −28.4% vs. baseline, miR-222: ∆ = −36% vs. baseline, miR-145: ∆ = +41.7% vs. baseline, all *p* < 0.001).

Comparisons between T0 and T1 are shown in Table 2, as well as T1 vs. controls comparisons. Of note, we found a significant change in miR expression after treatment; both miR-145 expression and miR-221/222 neared control values at T1, although remaining significantly different.

**Figure 2:**
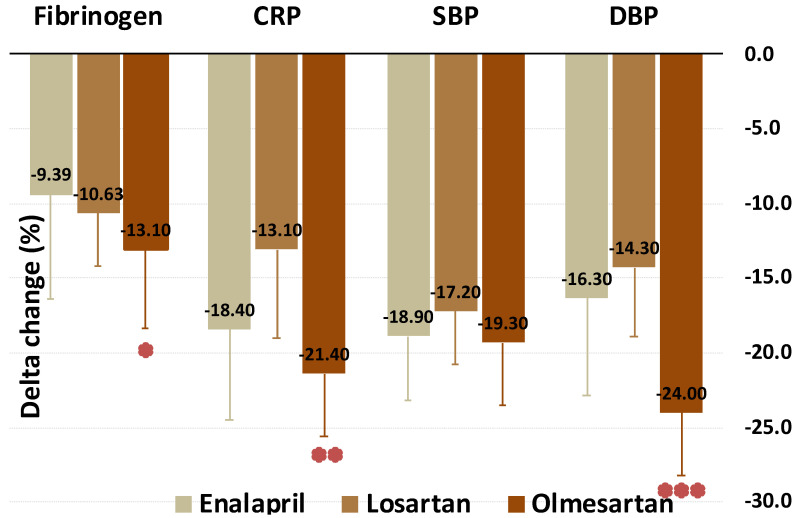
Change (%) of fibrinogen, CRP, SBP and DBP values by treatment with enalapril, losartan, and olmesartan. * Best, vs. enalapril; ** Best, vs. losartan; *** Best, vs. both enalapril and losartan.

**Figure 3:**
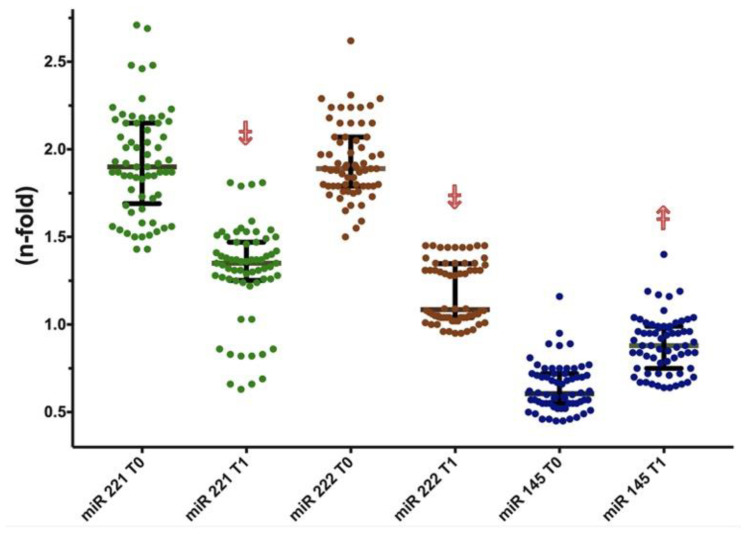

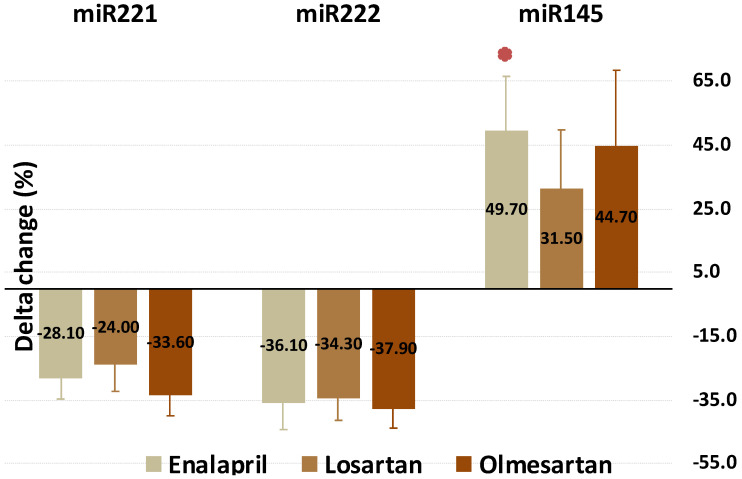
miR 221, 222 and 145 expression (upper panel) and change (%) from T0 to T1. Upper: n-fold expression with respect to basal healthy controls; ⤈/⤉: decrease/increase, *p* < 0.001 vs. T0. Lower: delta change (%) by treatment with enalapril, losartan, olmesartan. * Best, vs. losartan.

**Figure 4:**
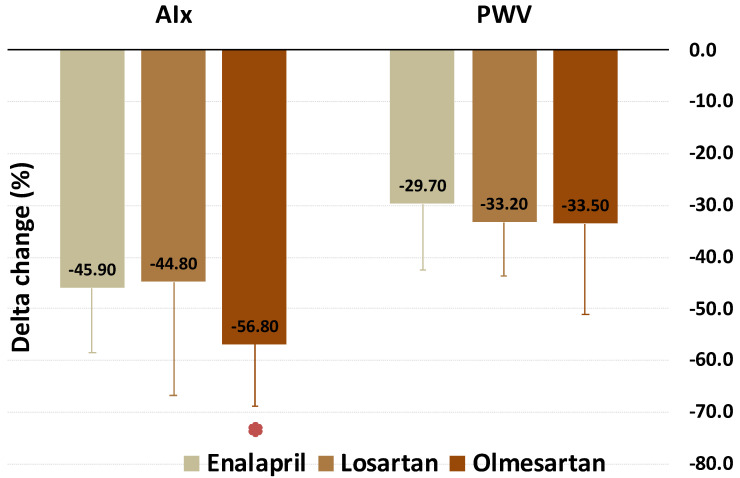
Change (%) of AIx and PWV by treatment with enalapril, losartan, and olmesartan. * Best, vs. both enalapril and losartan.

Furthermore, we compared the effects of the three pharmacological treatments on the considered variables. These results are shown in Figure 2, Figure 3 and Figure 4. Olmesartan was the most effective in reducing fibrinogen, DBP, CRP, and AIx (−13.1%, −19.3%, −21.4%, and −56.8%, respectively). Enalapril was the drug more significantly increasing the expression of miR-145. No difference was found as regards BMI, TC, TG, HDL-C, LDL-C, PWV, miRs–221/222. These data are summarized in Table 3.

Spearman’s correlations revealed the interrelationships between the variables: miRs 221 and 222 changes correlated with reduced SBP and DBP (both *p* < 0.001), with improved AIx and PWV (both *p* < 0.001), and also with reduced LDL-C and CRP (both *p* < 0.001); furthermore, we found that miR-145 correlated significantly with CRP (rs: 0.748), and inversely with SBP (rs:−0.789), DBP (rs: −0.768), AIx (rs: −0.599), PWV (rs: −0.652) (all *p* < 0.001).

Moreover, we also found that AIx and PWV improvement were significantly correlated with SBP and DBP reduction (both *p* < 0.001), as expected.

## 4. Discussion

Arterial hypertension is a chronic disease leading to a broad spectrum of complications, including atherosclerosis and myocardial infarction [35]. Endothelial dysfunction and the subversion of the artery wall are key features of hypertension [36,37]. Altered flow conditions modify vascular cell functions and induce changes in their transcriptional program, resulting in the release of a series of molecules and mediators that promote atherosclerosis and CVD [38,39].

The renin-angiotensin system (RAS) has a prominent role in the pathogenesis of hypertension and related organ damage [37,40]. Hence, angiotensin II receptor blockers and ACE-inhibitors are pivotal in the treatment of hypertension and cardiovascular disorders. In addition to lowering blood pressure, these drugs also have a significant impact in reducing CVD-associated morbidity and mortality [41]. In our previous study, we found that olmesartan, beyond lowering blood pressure, improved the inflammatory and lipid profile, and allowed a reduction of miR-221/222 levels in CD34+ CPCs from hypertensive patients with organ damage [9], suggesting that miR-221/222 decrease by olmesartan may also play a role in improving cIMT and in stabilizing carotid plaque [22,42].

The present study provides evidence for a correlation between arterial hypertension and changes in miR-145 and miR-221/222 levels in peripheral monocytes. In fact, we found altered expression of miRs in circulating monocytes from naïve hypertensive (at diagnosis) with respect to healthy controls; in particular, monocyte cells expressed increased levels of miR-221/222 and decreased levels of miR-145.

miR-221/222 are highly expressed in human ECs and are thought to regulate the development and functions of vascular endothelium [42,43]. Alexandru described that miRs have differential expression profiles in plasma, platelets, and platelet-derived microvesicles under hypertension associated with hyperlipidemia conditions. Additionally, the EPC-based therapy modified the expressions of these miRNAs in all three compartments [44]. In vitro studies showed that the knockdown of these miRs in ECs may alter pathways and molecules implicated in regulating EC functions, while the transfection of miR-221/222 mimics partially restored EC functions [43]. Further observations indicated that miR-221/222, by multiple target-mediated mechanisms, are able to maintain the quiescent state of ECs, preserving endothelial integrity and antiangiogenic properties [42]. Consistent with these data, ECs were reported to respond to vascular injury by induction of a proangiogenic transcriptional program, resulting in changes of miR-221/222 levels and EC phenotype [39]. Then, altered expression of miR-221/222 is critically involved in the development of arterial wall thickening and atherogenic disorders including hypertension [39,42]. Li et al. determined the plasma miR expression pattern of patients with essential hypertension and investigated the association and potential relationships among human cytomegalovirus (HCMV), hcmv–miR-UL112, and essential hypertension. They found increased HCMV seropositivity and quantitative titers in the hypertension group compared with the control group; in addition, they reported that copies of HCMV and hcmv–miR-UL112 were independently associated with an increased risk of hypertension [45].

It was noted that miR-221/222 seem to act towards vascular ECs and VSMCs in an opposite manner. In fact, while these miRs inhibit proliferation and migration and cause proapoptosis in ECs, both miRs stimulate proliferation and cell mobility and induce antiapoptosis in VSMCs [46]. Further, miR-221/222 induce switching from the contractile to synthetic phenotype in VSMCs, thus stimulating cell proliferation and mobility and promoting intima-media thickening and atherosclerotic vascular remodelling [42].

Moreover, miR-145 behaves differently in VSMCs and ECs: it is highly expressed in VSMCs, but almost undetectable in ECs [32]. Gene promoter of miR-145 contains binding sites for different transcriptional factors, regulating VSMC differentiation, plasticity and contractility [31,47]. Vascular injury promotes VSMC phenotypic switch from a contractile/non-proliferative to a migrating/proliferative state by inducing deregulation of miR-145 expression [47]; hence, downregulation of miR-145 results in VSMC dedifferentiation, proliferation, migration and production of extracellular matrix in neointimal tissue [47]. It was shown that miR-145 could be dramatically downregulated in balloon-injured rat carotid arteries; restoring miR-145 by viral transfection was able to inhibit neointimal lesion formation in rat carotid arteries after angioplasty [48]. A consistent finding was more recently reported also in human carotid artery stenosis [49]. Moreover, downregulation of miR-145 was also proposed as a marker of coronary event involving plaque rupture [50], reinforcing the concept that a balanced expression of this miR could be actively implicated in vascular health and stability.

Altogether, these evidence supports the hypothesis that a profile characterized by increased miR-221/222 and decreased miR-145 values could to be associated with a profibrotic and pro-atherogenic rearrangement.

Previously, we have shown that CRP, IL-6 and TNF-α plasma levels and monocyte biglycan expression in hypertensive subjects could be effectively reduced by blocking ATR1 with losartan [34]. Here, as a consequence of ATR1 blockade by losartan or olmesartan, or reduced AngII production due to ACE inhibition by enalapril, we found a significant improvement in miR expression in monocytes. Although miR-221/222 remained significantly highly expressed, these drugs seemed able to favourably modulate miR-221/222 and miR-145 expression, nearing their values to healthy controls, in addition to their effect on arterial hypertension lowering. This suggests that the use of RAAS modulators could be an effective strategy to reduce the pathological consequences of the complex rearrangement of ECs and VSMCs, classically referred to as negative vascular remodelling, as detectable by PWV, AS and cIMT assessment. Our data add new insights into the potential positive effect provided by a specific antihypertensive strategy in delaying atherogenesis in hypertensive patients. Furthermore, we could here suggest the use of miR-221/222 and miR-145 as potential therapeutic biomarkers, and likely as biomarkers for early detection of hypertensive individuals more prone to developing systemic complications [51].

## 5. Conclusions

We evaluated the effect of enalapril, losartan or olmesartan on miR profile in addition to their effect in blood pressure lowering and arterial stiffness indices’ improvement; we found that miR-221 and 222 significantly lowered, while miR-145 increased after a 24-week treatment. Moreover, we compared the effect of the different drugs; although no significant difference was found as regards BMI, TC, TG, HDL-C, LDL-C, PWV, and also miR-221/222 expression, we found that olmesartan was the most effective in reducing fibrinogen, DBP, CRP, and AIx, and enalapril the drug more significantly increasing the expression of miR-145. Notably, hypertensive subjects were prescribed to observe and maintain dietary and behavioural norms before and also after a drug was started. The blood sample at baseline was collected at the first visit, and then after 24 weeks of treatment also including behavioural norms. We cannot estimate the effect size of these measures. Altogether, these results suggest an overall improvement in the risk profile of hypertensive subjects after 24 weeks of treatment with enalapril, losartan or olmesartan, confirmed also by the balance among the miRs tested in the study.

Of course, these results should be considered with caution, also considering the small sample size, the lack of dependence analyses and of mechanistic implications, but if replicated on a larger scale, they could also indicate more specific effects within the class of RAAS inhibiting drugs beyond their anti-hypertensive effect, at least before non-reversible vascular damage was established.

## Figures and Tables

**Table 1:** Characteristics of study population (hypertensive patients and controls).

	Controls	Hypertensives (T0)	MW
Number	49	82	
Gender (m/f)	28/21	47/35	
	Median (IQR)		*p*
Age (years)	40 (13)	38 (31)	0.330
BMI (kg/m^2^)	24.7 (5.2)	25 (2.2)	0.424
SBP (mmHg)	120 (20)	155 (10)	<0.001
DBP (mmHg)	70 (10)	80 (10)	<0.001
TC (mg/dL)	178 (33)	175 (40)	0.892
HDL-C (mg/dL)	49 (11)	47 (6)	0.005
TG (mg/dL)	109 (22)	120 (31)	0.008
LDL-C (mg/dL)	107.2 (35.6)	104.6 (32.8)	0.643
Glucose (mg/dL)	88 (11)	88 (10.5)	0.802
HsCRP (mg/dL)	0.46 (0.29)	0.8 (0.15)	<0.001
Fibrinogen (mg/dL)	254.8 (84)	311 (55)	<0.001
AIx (%)	−3.9 (3.5)	13.3 (9.2)	<0.001
PWV (m/s)	4.9 (0.75)	8.12 (2.5)	<0.001
cIMT (mm)	0.77 (0.3)	0.91 (0.23)	<0.001
LVMi	71 (17.9)	74 (19)	0.358
miR-221 (n-fold)	0.97 (0.17)	1.9 (0.45)	<0.001
miR-222 (n-fold)	0.93 (0.13)	1.89 (0.27)	<0.001
miR-145 (n-fold)	1.04 (0.32)	0.61 (0.17)	<0.001

Data are presented as median (IQR). MW, Mann–Whitney test; *p*, significance level to statistic test to MW.

**Table 2:** Characteristics of hypertensive patients at baseline (T0) and after 6-month antihypertensive therapy (T1).

	Hypertensives (T0)	Hypertensives (T1)	Delta%	MWW T1 vs. T0	Controls	MWW T1 vs. C
Number	82				49	
Gender (m/f)	47/35				28/21	
	Median (IQR)			*p*		*p*
BMI (kg/m^2^)	25 (2.2)	24.17 (1.19)	−3.23	<0.001	24.7 (5.2)	0.573
SBP (mmHg)	155 (10)	125 (10)	−18.5	<0.001	120 (20)	0.001
DBP (mmHg)	80 (10)	70 (10)	−18.0	<0.001	70 (10)	0.314
TC (mg/dL)	175 (40)	170 (35)	−2.43	<0.001	178 (33)	0.310
HDL-C (mg/dL)	47 (6)	49 (4)	3.0	<0.001	49 (11)	0.132
TG (mg/dL)	120 (31)	120 (20)	−1.73	0.367	109 (22)	0.001
LDL-C (mg/dL)	104.6 (32.8)	99.5 (31.6)	−5.42	<0.001	107.2 (35.6)	0.200
Glucose (mg/dL)	88 (11)	87.5 (10)	−2.15	<0.001	88 (10.5)	0.196
HsCRP (mg/dL)	0.8 (0.15)	0.65 (0.19)	−17.5	<0.001	0.46 (0.29)	<0.001
Fibrinogen (mg/dL)	311 (55)	276 (63)	−11	<0.001	254.8 (84)	0.369
AIx (%)	13.3 (9.2)	6.8 (6.4)	−49.1	<0.001	−3.9 (3.5)	<0.001
PWV (m/s)	8.12 (2.5)	5.46 (2.1)	−32.2	<0.001	4.9 (0.75)	0.283
miR-221 (n-fold)	1.9 (0.45)	1.38 (0.23)	−28.4	<0.001	0.97 (0.17)	<0.001
miR-222 (n-fold)	1.89 (0.27)	1.20 (0.25)	−36.0	<0.001	0.93 (0.13)	<0.001
miR-145 (n-fold)	0.61 (0.17)	0.89 (0.22)	41.7	<0.001	1.04 (0.32)	<0.001

Data are presented as median (IQR). MWW, Mann–Whitney/Wilcoxon test; *p*, significance level to statistic test to MWW (T1 vs. T0 or T1 vs. Controls).

**Table 3:** Characteristics of hypertensive patients at baseline (T0) and after 6-month antihypertensive therapy (T1) according to the treatment assigned.

	Enalapril (T0)	Enalapril (T1)	Δ% *p* (MWW)	Losartan (T0)	Losartan (T1)	Δ% *p* (MWW)	Olmesartan (T0)	Olmesartan (T1)	Δ% *p* (MWW)	KW *
Number	27			29			26			
Gender (m/f)	20/7			21/8			20/6			
BMI (kg/m^2^)	24.4 (1.2)	24 (2)	−3.11 *p* < 0.001	25 (6)	25 (4)	−2.83 *p* = 0.001	25.3 (3.4)	25 (2.27)	−3.78 *p* < 0.001	*p* = 0.740
SBP (mmHg)	155 (10)	120 (10)	−18.9 *p* < 0.001	160 (15)	130 (18)	−17.2 *p* < 0.001	150 (11)	120 (5)	−19.3 *p* < 0.001	*p* = 0.089
DBP (mmHg)	80 (5)	70 (0)	−16.3 *p* < 0.001	85 (10)	70 (10)	−14.3 *p* < 0.001	80 (10)	60 (10)	−24 *p* < 0.001	*p* < 0.001
TC (mg/dL)	169 (22)	170 (30)	−2.36 *p* = 0.007	180 (40)	180 (35)	−2.63 *p* < 0.001	165 (44)	160 (33)	−2.3 *p* = 0.01	*p* = 0.974
HDL-C (mg/dL)	46 (6)	48 (4)	3.9 *p* < 0.001	45 (9)	47 (5)	2.14 *p* = 0.009	48 (2)	49 (1)	3.32 *p* = 0.02	*p* = 0.316
TG (mg/dL)	120 (31)	120 (19)	4.1 *p* = 0.273	120 (18)	113 (10)	0.51 *p* = 0.927	120 (37)	120 (29)	0.66 *p* = 0.821	*p* = 0.352
LDL-C (mg/dL)	101 (29.4)	93 (33.8)	−6.6 *p* < 0.001	113 (40.3)	108 (32)	−4.7 *p* < 0.001	93.1(41.3)	84 (31.5)	−4.9 *p* = 0.007	*p* = 0.542
Glucose (mg/dL)	85 (11)	90 (10)	0.31 *p* = 0.876	86 (12)	80 (10)	−3.6 *p* = 0.002	89 (11)	88 (10)	−3.1 *p* = 0.008	*p* = 0.008
HsCRP (mg/dL)	0.82 (0.11)	0.69 (0.18)	−18.4 *p* < 0.001	0.82 (0.12)	0.73 (0.15)	−13.1 *p* < 0.001	0.7 (0.23)	0.54 (0.21)	−21.4 *p* < 0.001	*p* = 0.002
Fibrinogen (mg/dL)	302.8 (41.2)	295 (62)	−9.39 *p* < 0.001	324 (33.4)	290 (37)	−10.63 *p* < 0.001	287.2 (72.3)	242.4 (45)	−13.1 *p* < 0.001	*p* = 0.018
AIx (%)	12.8 (5.3)	7 (3.3)	−45.9 *p* < 0.001	16.2 (9.2)	7 (7.8)	−44.8 *p* < 0.001	11.1 (7.1)	4 (4.8)	−56.8 *p* < 0.001	*p* = 0.007
PWV (m/s)	8 (2.3)	5.6 (2.2)	−29.7 *p* < 0.001	8.6 (1.6)	5.4 (2.1)	−33.2 *p* < 0.001	7.4 (2.1)	4.4 (1.0)	−33.5 *p* < 0.001	*p* = 0.491
miR-221 (n-fold)	1.87 (0.74)	1.35 (0.06)	−28.1 *p* < 0.001	2.05 (0.32)	1.5 (0.17)	−24 *p* < 0.001	1.64 (0.7)	1.11 (0.76)	−33.6 *p* < 0.001	*p* = 0.055
miR-222 (n-fold)	1.89 (0.08)	1.27 (0.29)	−36.1 *p* < 0.001	2.06 (0.42)	1.33 (0.35)	−34.3 *p* < 0.001	1.8 (0.19)	1.18 (0.05)	−37.9 *p* < 0.001	*p* < 0.634
miR-145 (n-fold)	0.62 (0.15)	0.98 (0.15)	49.7 *p* < 0.001	0.57(0.1)	0.75 (0.17)	31.5 *p* < 0.001	0.68(0.22)	0.95 (0.11)	44.7 *p* < 0.001	*p* = 0.001

Data are presented as median (IQR). MWW, Mann–Whitney/Wilcoxon test; *p*, significance level to statistic test to MWW (T1 vs. T0) KW, Kruskal–Wallis test; *p*, significance level to statistic test to KW. * significant *p*-values were tested by two-by-two comparisons with Bonferroni correction; for details see Table 4.

**Table 4:** Two-by-two comparisons by treatment.

	Enalapril	Losartan	Olmesartan	*p*
Fibrinogen	Worst	-	Best	=0.015
CRP	-	Worst	Best	<0.001
DBP	Worst	Worst	Best	<0.001
miR-145	Best	Worst	-	<0.001
AIx	Worst	Worst	Best	<0.05

Significant *p*-values from KW test were tested by two-by-two comparisons with Bonferroni correction. The table shows the best/worst from the comparisons, with its statistical level.

## Data Availability

All data generated or analyzed during the study are included in this published article.

## Abbreviations

Aix	augmentation index
AS	arterial stiffening
ATR1	angiotensinII-type1 receptor
ARB	angiotensinII-type1 receptor blocker
BMI	body mass index
BNP	B-type natriuretic peptide
BSA	body surface area
c-IMT	carotid intima media thickness
CPCs	circulating progenitor cells
CRP	C-reactive protein
CV	cardiovascular
CVD	cardiovascular disease
CVE	cardiovascular events
DBP	diastolic blood pressure
ECs	endothelial cells
EF	ejection fraction
ESR	erythrocyte sedimentation rate
HDL	high-density lipoprotein
HF	heart failure
HR	heart rate
HRT	hormone-based therapy
IQR	interquartile range
KW	Kruskal-Wallis
LDL-C	low-density-lipoprotein-cholesterol
LVH	left ventricular hypertrophy
LVMI	left ventricular mass index
MWW	Mann-Whitney/Wilcoxon test
Mirs	microRNAs
ncRNA	non-coding RNA
PWV	pulse-wave velocity
ROS	reactive oxygen species
SBP	systolic blood pressure
TC	Total cholesterol
US	ultra-sonography
VSMCs	vascular smooth cells
